# Dispersal without drivers: Intrinsic and extrinsic variables have no impact on movement distances in a terrestrial amphibian

**DOI:** 10.1002/ece3.9368

**Published:** 2022-10-01

**Authors:** Nathalie Jreidini, David M. Green

**Affiliations:** ^1^ Department of Biology McGill University Montréal Quebec Canada; ^2^ Redpath Museum McGill University Montréal Quebec Canada

**Keywords:** amphibian, density‐dependence, displacement, landscape dynamics, movement ecology, sex‐bias

## Abstract

Dispersive movements are often thought to be multicausal and driven by individual body size, sex, conspecific density, environmental variation, personality, and/or other variables. Yet such variables often do not account for most of the variation among dispersive movements in nature, leaving open the possibility that dispersion may be indeterministic. We assessed the amount of variation in 24 h movement distances that could be accounted for by potential drivers of displacement with a large empirical dataset of movement distances performed by Fowler's Toads (*Anaxyrus fowleri*) on the northern shore of Lake Erie at Long Point, Ontario (2002–2021, incl.). These toads are easy to sample repeatedly, can be identified individually and move parallel to the shoreline as they forage at night, potentially dispersing to new refuge sites. Using a linear mixed‐effect model that incorporated random effect terms to account for sampling variance and inter‐annual variation, we found that all potential intrinsic and extrinsic drivers of movement accounted for virtually none of the variation observed among 24 h distances moved by these animals, whether over short or large spatial scales. We examined the idea of movement personality by testing variance per individual toad and found no evidence of individuality in movement distances. We conclude that deterministic variables, whether intrinsic or extrinsic, neither can be shown to nor are necessary to drive movements in this population over all spatial scales. Stochastic, short time‐scale movements, such as daily foraging movements, can instead accumulate over time to produce large spatial‐scale movements that are dispersive in nature.

## Graphical abstract

Dispersive movements are often thought to be multicausal. In this study, we assessed the amount of variation in 24 h movement distances that could be accounted for by pontial drivers of displacement using 20 years of data on Fowler's Toads (*Anaxyrus fowleri*). We conclude that movements in this population, over all spatial scales, cannot be explained by deterministic variables. Short stochastic movements can instead accumulate over time to produce dispersive movements.

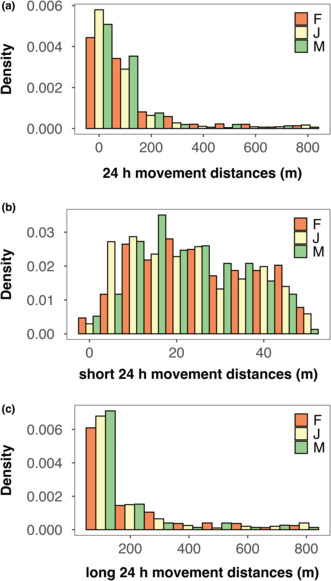

“Animal dispersal is on the whole a rather quiet, humdrum process, […] taking place all the time as a result of the normal life of the animals.”—Charles Elton, Animal Ecology (1927)

## 1 INTRODUCTION

Organismal movements are considered crucial components of population and community dynamics, whether these movements occur as part of an organism's life history or arise in response to environmental variables. Dispersal, the displacement of individual organisms that could lead to gene flow (Marsh & Trenham, 2001; Ronce, 2007), is especially of central importance to population ecology and evolution. On one hand, because dispersal is necessary for effects ranging from outbreeding to geographic range expansion, it is generally acknowledged as beneficial for most populations and therefore, with only rare exceptions, selectively advantageous (Hamilton & May, 1977; Johnson & Gaines, 1990; Parvinen et al., 2003; Poethke et al., 2003). On the other hand, dispersal by individuals away from habitable localities, without guarantee of finding another habitable site, is a highly risky endeavor, often with very low odds of success (Bonte et al., 2012; Clobert et al., 2001; Cote & Clobert, 2010; Stamps, 2001). This contradiction is a fundamental problem for understanding the ecology of dispersal. What drives individual organisms to disperse?

For many organisms, the answer to this question is straightforward—they have no say in whether they disperse or not. Propagules of sessile organisms, such as most plants, cannot occupy the same physical spaces as their parents and therefore must somehow disperse away. The seeds themselves are entirely passive when it comes to their own dispersal, and their dispersive trajectories may largely be stochastic (Nathan et al., 2011). Most animals, though, are motile at all life stages, and thus a variety of variables may exist to compel individuals to disperse under their own power (Bowler & Benton, 2005; Matthysen, 2012).

Many suggested drivers of dispersal are intrinsic properties of the animals themselves. Sex‐biased dispersal, for instance, is widely reported among animals and may be related to mating systems (Greenwood, 1980) and/or the distribution of critical resources, such as nesting sites or potential mates (Li & Kokko, 2019). However, if both sexes are equally affected by the distribution of resources, dispersal should not be sex‐biased (Johnson & Gaines, 1990) as seen in certain birds (Mäki‐Petäys et al., 2007) and amphibians (Berven & Grudzien, 1990; Sinsch et al., 2012; Trenham et al., 2001). Age and body size may also affect dispersal tendencies in that larger, older individuals may be able to move further than smaller individuals (Choi et al., 2003; Jenkins et al., 2007; Phillips et al., 2006) or, conversely, outcompete smaller individuals and so push them to move away (Bowler & Benton, 2005). Individual behavioral differences in boldness or inquisitiveness (Dall et al., 2004; Fraser et al., 2001; Nilsson et al., 2014; Sih et al., 2004), often referred to as “personalities”, may also affect movement behavior. The existence of movement personalities has been documented in certain lizards (Cote & Clobert, 2007), birds (Kurvers et al., 2009; Minderman et al., 2009), fishes (Kobler et al., 2009), and even amphibians (Kelleher et al., 2018). Investigations into amphibian personalities have been concerned with three behavioral syndromes: boldness, exploration, and activity (Kelleher et al., 2018). Exploration behavior has been shown to be positively correlated with dispersive patterns in the invasive cane toads (*Rhinella marina*; Gruber et al., 2017). Additional studies on anuran activity have been largely restricted to the larval stage (e.g., Urszán et al., 2015) and, in one case, extended to metamorphosis (Wilson & Krause, 2012). As dispersal typically takes place post‐metamorphosis and movement patterns differ in aquatic versus terrestrial habitats, it remains unclear the extent to which personalities can drive movement patterns of adult terrestrial amphibians within and between habitats (Kelleher et al., 2018).

There are other potential drivers of dispersal that are instead properties of the environment extrinsic to the individual animals. Conspecific density, leading to varying levels of intraspecific competition (Baguette et al., 2011; Bowler & Benton, 2005; Clobert et al., 2004; Ronce, 2007) has been positively correlated with dispersal in numerous fishes (Connor et al., 2013; Taylor et al., 2013), reptiles (Vignoli et al., 2012), birds (Molina‐Morales et al., 2012; Pärn et al., 2012), and amphibians (Ousterhout & Semlitsch, 2018). However, if living in groups is selectively advantageous, as seen in certain fruit flies (Betini et al., 2015), amphibians (Cayuela et al., 2019), mammals (Denomme‐Brown et al., 2020; Mabry, 2014), birds (Forero et al., 2002; Fuentes et al., 2019; McKellar et al., 2015), or reptiles (Calsbeek, 2009), conspecific density can, instead, be negatively correlated with dispersal. Complicating matters further, sex‐biased dispersal patterns may appear in response to density (De Bona et al., 2019; Fattebert et al., 2015; Scandolara et al., 2014), making dispersal potentially density‐dependent and, to a certain extent, context dependent (Bocedi et al., 2012; Cayuela et al., 2018).

Landscape dynamics are also suggested to impact the displacements of animals (Morales et al., 2010). Landscapes with little variation in either structure or resources should promote range residency and thus lead to a smaller range of movement distances, as seen in ungulate populations (Mueller et al., 2011), whereas landscapes that vary unpredictably lead to unpredictable movement trends, as has been reported in Eurasian red squirrels, for example (Hämäläinen et al., 2019). As such, irregular movements that are difficult to predict and are neither migratory nor philopatric have been referred to as “nomadic” and have been associated with environments that are highly variable, both spatially and temporally (Jonzén et al., 2011; Mueller & Fagan, 2008; Mueller et al., 2011; Singh et al., 2012; Teitelbaum & Mueller, 2019).

Amphibians can offer several advantages over many other types of animals for studying movement dynamics. Even their largest movements are relatively small enough to be readily detectable (Smith & Green, 2005) and numerous amphibian populations are amenable to being monitored in long‐term studies over many consecutive years (e.g., Cayuela et al., 2019; Sinsch, 2014). In particular, the Fowler's Toads (*Anaxyrus fowleri*) found at Long Point, Ontario, Canada, a 35 km long sand spit on the northern shore of Lake Erie, represent a study system that is especially well‐suited for investigating dispersive movements in relation to potential drivers of dispersal (Smith & Green, 2006). The movements made by these toads are almost entirely restricted to a sandy beach running east–west, parallel to the lakeshore, making such displacements essentially one‐dimensional, though potentially subject to variations in lake water level that can alter the extent and structure of the beach. The toads' diel activity pattern is almost entirely nocturnal, and Marchand et al. (2017) found that whether they returned to previously occupied daytime refuge sites or found new ones was largely stochastic. Furthermore, the toads are easily and repeatably captured while they are active at night and are readily identifiable as individuals (Schoen et al., 2015), making it possible to amass a large dataset of individualized movement distances. If any particular variables are drivers of the movements made by these toads, then they should account for significant amounts of the variation seen in the toads' movement distances, especially long‐distance movements. Alternatively, if none of these variables significantly drive the toads' movements, then dispersal in this population could be said to arise as the accumulation of indeterministic movements.

## 2 METHODS

### 2.1 Data collection and study system

We used the dataset of geo‐referenced (NAD 83 Datum) captures of individually identified toads amassed by Smith and Green (2006) over 4 years (2002–2005, incl.) and augmented it with equivalent data gathered over a further 16 years (2006–2021, incl.). The study site was an 8.3 km stretch of shoreline consisting of beaches, dunes, marshlands, and areas of settlement (Greenberg & Green, 2013; Smith & Green, 2005). Unlike previous surveys of this species (Smith & Green, 2006), we did not toe‐clip animals for identification. Instead, we identified individuals based on their unique patterns of dorsal spots in photographs (Schoen et al., 2015), which enabled us to assign every individual, including juveniles, a unique identity number (toad ID) and track them throughout the active season and from year to year. We identified individuals as either juvenile, adult male, or adult female based on SVL (snout‐to‐vent length, mm) and throat color (see Smith & Green, 2006).

We used the UTM geo‐coordinates to calculate Euclidean distances, in meters, between successive encounters of individual toads on the beach. We restricted the dataset to observations on the beach, where all individuals had equal access to the water source (Lake Erie), to exclude springtime migratory movements made by adult toads to and from breeding sites in adjacent marshes and ponds (Marchand et al., 2017). We considered movement distances irrespective of their directionality. To derive a measure of the toads' density on any given occasion, we used the UTM geo‐coordinates to calculate nearest‐neighbor distance between individual toads active on the same night.

We obtained data on Lake Erie water levels, in meters above mean sea level, from the website of the US Army Corps of Engineers, Detroit District (https://www.lre.usace.army.mil/) and daily weather conditions (total daily precipitation, in millimeters, and mean daily air temperature, in °C) from the Environment Canada website (https://climate.weather.gc.ca/) for the Port Colborne, Ontario, weather station.

### 2.2 Analysis

All calculated distances, in meters, were log_10_‐transformed before analysis to reduce skewness. We used ANOVA to first test whether movement distance was correlated with lag time (i.e., the time elapsed between encounters, in days). As there was a significant correlation (*F*_1, 6252_ = 211.1, *p* < .001), subsequent analyses were carried out on the subset of movement distances that occurred during 24 h (i.e., encountered on consecutive nights) to remove any biases resulting from variation in lag time between successive encounters (Blouin‐Demers & Weatherhead, 2021; Gamble et al., 2007).

As *A. fowleri* are size‐dimorphic, with female adults larger than both adult males and juveniles, we first tested for a variation in *SVL* (proxy for body size) between sexes using ANOVAs. We then tested for a sex bias in movement distances with a subset of the dataset to include adult males and adult females of comparable size. Similarly, we tested for an age‐bias with a subset of the dataset to include adult males and juveniles of the same size range, as adult females and juvenile sizes do not overlap. We used linear mixed‐effect models (LMMs) on these subsets, with fixed effect terms *sex* (categorical with 3 levels: adult males, adult females, and juveniles) and *SVL* (continuous, in mm). Random effect terms *year* and *toad ID* (the individual identities of the animals) were included in all LMMs to account for inter‐annual and inter‐individual sampling variance, respectively.

We then used LMMs to assess the impact of intrinsic and extrinsic drivers on movement distances performed by the toads. The intrinsic categorical term, *sex*, which included both sexually mature adults as well as pre‐sexual juveniles, was added to the LMM as an interaction term with the continuous term *SVL*. Extrinsic, continuous fixed effect terms added to the LMM were *nearest‐neighbor distance* (log_10_‐transformed), *air temperature* (mean daily temperature), *precipitation* (total daily rainfall), and *lake level* (mean daily water level). *Air temperature* and *precipitation* were added as terms for the day of encounter (time *t*) and for the day prior to encounter (time *t* − 1). Lake level does not vary enough from day to day to justify adding two time‐points. Instead, we added a parameter for changes in annual mean lake level, referred to as *landscape variability* since Lake Erie directly impacts the extent of the toads' habitat on the beach. Desiccation risk was not deemed worth investigating as individuals of this population have unrestricted access to the lake for hydration. We obtained parameter estimates using maximum likelihood with the Laplace approximation method (Bates et al., 2020). We also inferred the intraclass correlation coefficient (ICC) to further test whether movement distances were nested per *year* and/or per *toad ID*, whereby an ICC value below .50 would suggest low similarity within a year and/or within an individual (Koo & Li, 2016).

To determine the model that explains the greatest amount of variation in the response variable using the fewest number of independent variables, we compared conditional Akaike information criterion (cAIC) using R package ‘cAIC4’ (Säfken et al., 2021) and report the Akaike weights of the best‐fit models. We report *R* ^2^ values as coefficients of determination in linear regressions, marginal *R* ^2^ values to represent variation accounted for by all fixed effect variables, and conditional *R* ^2^ values to represent variation accounted for by all fixed and random effect variables in LMMs (Nakagawa & Schielzeth, 2013). Multicollinearity in the regression models was examined by obtaining variance‐inflation factors (VIF), where a VIF value of around 1 indicates no correlation between predictor variables, and a value greater than 5 indicates a strong correlation between predictor variables and would need to be considered (Fox & Weisberg, 2019).

To distinguish between 24 h movements within close proximity to refuge sites versus ones that consist of a more prominent net displacement, we considered distances below the median distance of the whole dataset of 24 h movement distances to be “short 24 h distances”, and distances above the median to be “long 24 h distances”. We repeated the LMM analysis described above on the two data subsets. We assessed normality in the distribution of all data subsets using the Shapiro–Wilk test.

To test for individual movement personalities among toads, we looked for evidence of consistency in the magnitude of distances moved by individuals. Specifically, we asked whether long‐distance movements were prevalent only among certain individuals or randomly distributed among all individuals. To do this, we used standard deviation as our linear measure of the variability among distances moved by individuals. We reasoned that if individuals had distinct movement personalities, then the within‐individual standard deviations should be relatively low, showing consistency, and between‐individual standard deviations should be significantly lower than expected if movement distances were distributed at random among all individuals. Accordingly, we calculated the standard deviations among 24 h movement distances for each individual toad encountered at least 5 times during a field season and then calculated the standard deviation of those standard deviations. We then randomized the data 1000 times and, each time, made the same calculations. This yielded a distribution of 1000 values to compare against the actual value using Fisher's exact test, and allowed us to assess whether our actual value falls within the distribution obtained for randomized values. To test that the concept of this method of analysis was valid, we also sorted the distance data from largest to smallest, and from smallest to largest, to produce two non‐random datasets and computed standard deviations, as above, for comparison to the set of random‐derived values. For this analysis only, we augmented our capture‐mark‐recapture dataset with comparable 24 h movement data derived from radio‐tracking studies of the same population of *A. fowleri* conducted in 2007 (N. Sanderson and D. M. Green *unpublished*), 2008 (Green & Yagi, 2018), 2009 and 2010 (Marchand et al., 2017).

All statistical analyses, randomizations, and visualizations were done in R version 3.6.3 (R Core Team, 2021). LMMs were conducted using R packages ‘lme4’ (Bates et al., 2020) and ‘MuMIn’ (Bartoń, 2020).

## 3 RESULTS

We amassed a dataset of 6254 displacement distances for 1443 individual toads over 20 years (2002–2021, incl). Lag times ranged from 1 to 86 days. The total dataset was comprised of observations for 700 adult females, 393 adult males, and 842 juveniles, whereby juveniles were identified as either female or male once sexually mature. As is invariably the case for dispersal data (Fraser et al., 2001; Smith & Green, 2006) and for short time‐scale movements in this population (Jreidini & Green, 2022), the frequency distribution of toad movement distances was right‐skewed, for distances over varying lag times (*x̃* = 60.08 m, *x̄* = 172.57 m; Shapiro–Wilk test: *W* = 0.60, *p* < .001).

**FIGURE 1:**
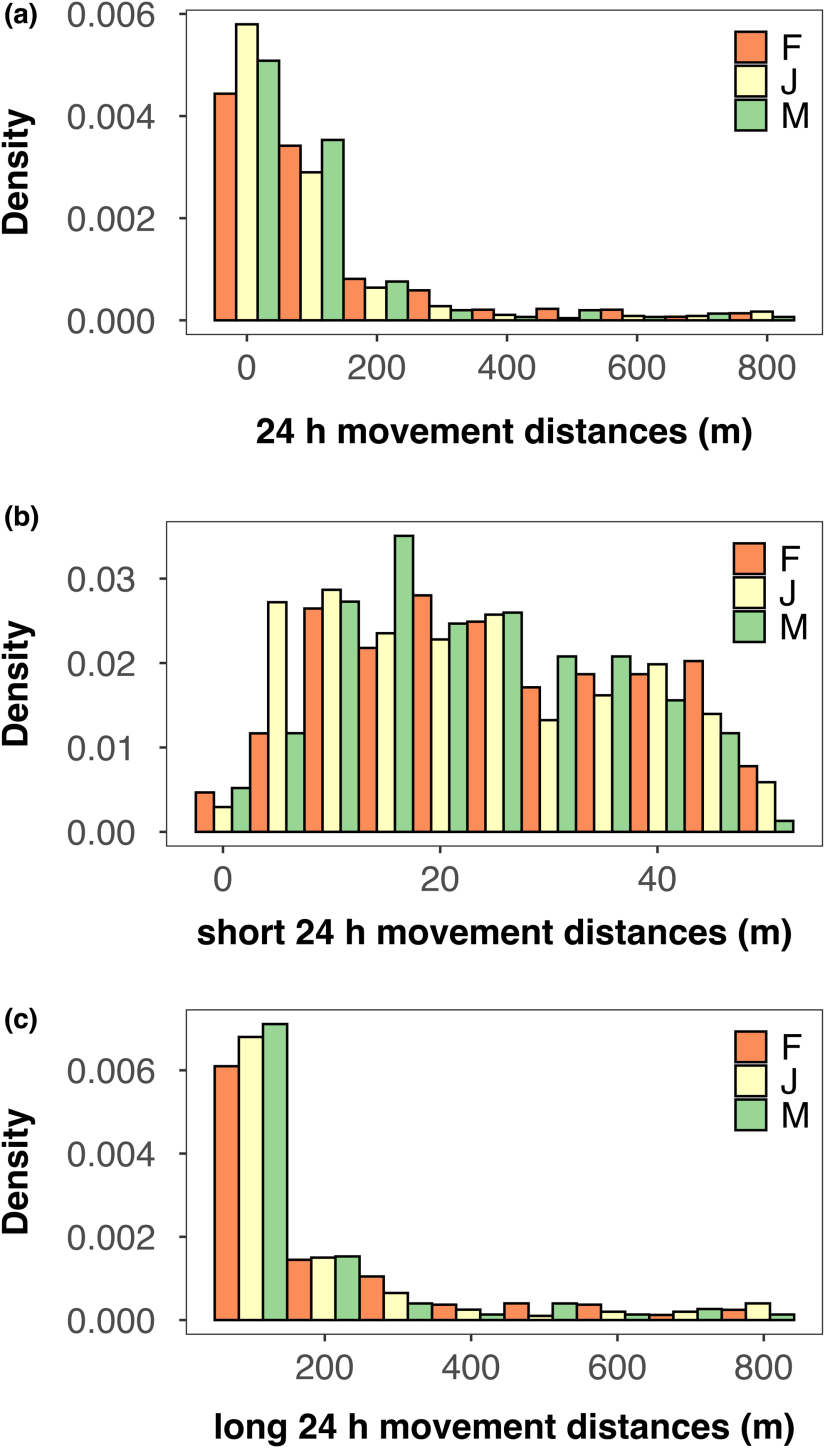
Probability distribution of untransformed movement distances for (a) all 24 h distances in 100 m bins (*n* = 1365) and (b) short 24 h distances in 5 m bins (*n* = 683) and (c) long 24 h distances in 50 m bins (*n* = 682) performed by individual Fowler's Toads, *Anaxyrus fowleri*, at Long Point, Ontario (2002–2021, incl). F, adult female; J, juvenile; M, adult male.

The log–log regression between displacement distances and lag time had little explanatory power (*R* ^2^ = .032), despite its high significance (ANOVA: *F*_1, 6252_ = 211.1, *p* < .001), justifying the restriction of our analyses to only the subset of 24 h movement distances to eliminate the influence of lag time. This subset of “all 24 h distances” thus consisted of 1365 displacements undertaken over 24 h for 707 individuals (*x̃* = 49.50 m, *x̄* = 100.98 m) and had a right‐skewed distribution as well (Shapiro–Wilk statistic: 0.62, *p* < .001; Figure 1a). From this dataset, we obtained the subset of “short 24 h distances” with 683 displacements (*x̃* = 22.47 m, *x̄* = 23.75 m; Shapiro–Wilk test: *W* = 0.96, *p* < .001; Figure 1b) and the subset of “long 24 h distances” with 682 displacements (*x̃* = 105.46 m, *x̄* = 178.33 m; Shapiro–Wilk test: *W* = 0.71, *p* < .001; Figure 1c).

We found that 24 h movement distances were neither sex‐biased nor age‐biased, taking differences in *SVL* into consideration. Average *SVL* differed greatly among males, females, and juveniles (ANOVA: *F*_2, 6251_ = 6227, *p* < .001, *R* ^2^ = .665), with adult females 65.77 ± 6.69 mm on average, adult males 57.56 ± 4.92 mm on average, and sexually immature juveniles 44.93 ± 6.23 mm, on average. However, the difference in movement distances between adult males and adult females of the same size range (55–75 mm) was not significant (ANOVA: *F*_2, 733_ = 2.42, *p* = .090, *R* ^2^ = .004), even when accounting for random effect terms *year* and *toad ID* (LMM: Estimate = 0.014, *SE* = 0.010, *t* = 1.451, *p* = .147). Likewise, the difference in movement distances between adult males and juveniles of the same size range (40–55 mm) was also not significant (ANOVA: *F*_1, 475_ = 0.038, *p =* .847, *R* ^2^ = .002; LMM: Estimate = −0.0001, *SE* = 0.015, *t* = −0.059, *p* = .953).

The best‐fit LMMs for “all 24 h distances” and “long 24 h distances” based on cAIC included all fixed and random effect variables (cAIC_all_ = 4278; cAIC_long_ = 1510) and carried 57% and 99% of the cumulative model weight, respectively. The best‐fit LMM for “short 24 h distances” only included the interaction between fixed effect variables *sex* and *SVL*, and random effect variable *toad ID* (cAIC_short_ = 1543), carrying 73% of the cumulative model weight. Nonetheless, the full models, which included all fixed and random effect variables, were reported to show the effects of all potential drivers for each response variable (Table 1). VIF values showed a lack of collinearity between predictor variables in all LMMs (VIF ≈ 1) except for *SVL* and *sex* (VIF > 5), further supporting their addition as an interaction term in LMMs.

**TABLE 1:** LMM coefficients for the full model for log_10_‐transformed response variables 24 h movement distances (*n* = 1365), short 24 h movement distance (*n* = 683), and long 24 h movement distance (*n* = 682) performed by Fowler's Toads, *Anaxyrus fowleri* (*N* = 713) at Long Point, Ontario (2002–2021, incl). Intrinsic fixed effect variables: *SVL* is the snout‐to‐vent length (in mm), and *sex* corresponds to both sex at maturation and age (adult female, adult male, or juvenile). Extrinsic fixed effect variables: *Nearest‐neighbor distance* (log_10_‐transformed) is the distance to the nearest toad at that encounter, *air temperature* corresponds to the daily mean ambient temperature (°C) and *precipitation* corresponds to the total daily rainfall (mm) both at time of encounter (*t*) and 1 day prior to encounter (*t* − 1). *Lake level* corresponds to the daily mean water level (m) and *landscape variability* corresponds to the shift in water level from annual mean. Random effect variables *toad ID* and *year* are included in all LMMs.

Response variable	Fixed effect variables	Estimate	CI^a^	*t*	*p*	*R* ^2^
log_10_(all 24 h distances)	Intercept (full model)	44.349	−28.739 to 117.203	1.108	.268	.034
	SVL	−0.009	−0.025 to 0.008	−1.039	.299	.001
	SVL × Sex (M)	0.056	0.022 to 0.088	3.281	.030*	.009
	SVL × Sex (J)	0.027	0.003 to 0.052	2.167	.001**	.004
	log_10_(Nearest‐neighbor distance)	0.019	−0.003 to 0.035	2.272	.023*	.004
	Air temperature_(*t*)_	−0.000	−0.035 to 0.031	−0.012	.990	.000
	Air temperature_(*t*−1)_	−0.001	−0.027 to 0.027	−0.044	.965	.000
	Precipitation_(*t*)_	−0.008	−0.022 to 0.007	−1.044	.297	.001
	Precipitation_(*t*−1)_	−0.002	−0.014 to 0.009	−0.381	.703	.000
	Lake level	−0.229	−0.648 to 0.191	−0.995	.320	.003
	Landscape variability	1.160	0.020 to 2.454	1.900	.050*	.007
log_10_(short 24 h distances)	Intercept (full model)	9.052	−29.641 to 47.603	0.455	.649	.037
	SVL	−0.006	−0.020 to 0.008	−0.773	.439	.001
	SVL × Sex (M)	0.026	−0.003 to 0.054	1.790	.073	.005
	SVL × Sex (J)	0.018	−0.002 to 0.039	1.719	.086	.004
	log_10_(Nearest‐neighbor distance)	−0.000	−0.015 to 0.013	−0.078	.938	.000
	Air temperature_(*t*)_	−0.041	−0.069 to 0.013	−2.833	.005**	.012
	Air temperature_(*t*−1)_	0.018	−0.005 to 0.041	1.490	.136	.003
	Precipitation_(*t*)_	0.005	−0.009 to 0.020	0.741	.458	.001
	Precipitation_(*t*−1)_	−0.006	−0.016 to 0.004	−1.199	.231	.002
	Lake level	−0.030	−0.252 to 0.193	−0.262	.794	.000
	Landscape variability	0.369	−0.307 to 1.044	1.060	.289	.002
log_10_(long 24 h distances)	Intercept (full model)	64.870	−3.118 to 132.396	1.763	.078	.054
	SVL	0.000	−0.013 to 0.013	0.008	.994	.000
	SVL × Sex (M)	0.025	−0.004 to 0.053	1.710	.087	.004
	SVL × Sex (J)	−0.026	−0.048 to −0.004	−2.372	.018*	.008
	log_10_(Nearest‐neighbor distance)	0.011	−0.003 to 0.025	1.492	.136	.003
	Air temperature_(*t*)_	0.019	−0.010 to 0.049	1.288	.198	.003
	Air temperature_(*t*−1)_	−0.009	−0.032 to 0.015	−0.719	.472	.001
	Precipitation_(*t*)_	−0.017	−0.029 to 0.006	−2.876	.004**	.012
	Precipitation_(*t*−1)_	−0.004	−0.013 to 0.007	−0.645	.519	.001
	Lake level	−0.346	−0.734 to 0.045	−1.636	.102	.015
	Landscape variability	0.882	−0.146 to 1.954	1.585	.113	.010

Abbreviations: LMM, linear mixed‐effect model; SVL, snout‐to‐vent length.

^a^ 2.5%–97.5% confidence intervals.

* Statistically significant at *α* = .05.

** Statistically significant at *α* = .01.

A few fixed effect variables were found to be statistically significant, but they had little explanatory power (Table 1). *Nearest‐neighbor distance*, a proxy for conspecific density, and the interaction between *sex* and *SVL* were found to be significant (*p* < .05) yet weak (Estimates < 1, *R* ^2^ < .01) positive predictors of movement distances, but only for “all 24 h distances”. The interaction between *sex* and *SVL* was found to be a significant yet weak negative predictor for “long 24 h distances”, but only in juveniles (Estimate = −0.026, *p* < .05, *R* ^2^ = .008). For environmental variables, results differed for each data subset. *Landscape variability* was significantly positively correlated with “all 24 h distances” (Estimate = 1.160, *p* < .01) although it did not explain much of the variation (*R* ^2^ = .007), and the effect was no longer significant in the data subsets for short and long distances (*p* > .05). *Air temperature* (time *t*) was significantly negatively correlated with “short 24 h distances” (Estimate = −0.041, *p* < .01, *R* ^2^ = .012) while *precipitation* (time *t*) was significantly negatively correlated with “long 24 h distances” (Estimate = −0.017, *p* < .05, *R* ^2^ = .008). Nonetheless, as correlation estimates and coefficients were all negligible, the statistical significance can be ascribed to the large statistical power of such a large dataset.

The variation in movement distances accounted for by all potential drivers, or fixed effect variables, in each full model was negligible according to marginal *R* ^2^ values ($R_{\mathrm{all}}^{2}$ = .033; $R_{\text{short}}^{2}$ = .037; $R_{\text{long}}^{2}$ = .052). Similarly, random effect variables only accounted for a small portion of the variation according to conditional *R* ^2^ values ($R_{\mathrm{all}}^{2}$ = .208; $R_{\text{short}}^{2}$ = .107; $R_{\text{long}}^{2}$ = .216). Moreover, the ICC was low for “all 24 h distances” (ICC = .18), for “short 24 h distances” (ICC = .07), and for “long 24 h distances” (ICC = .17) suggesting that the variation in movement distances is not significantly nested either per *year* or per *toad ID*.

**FIGURE 2:**
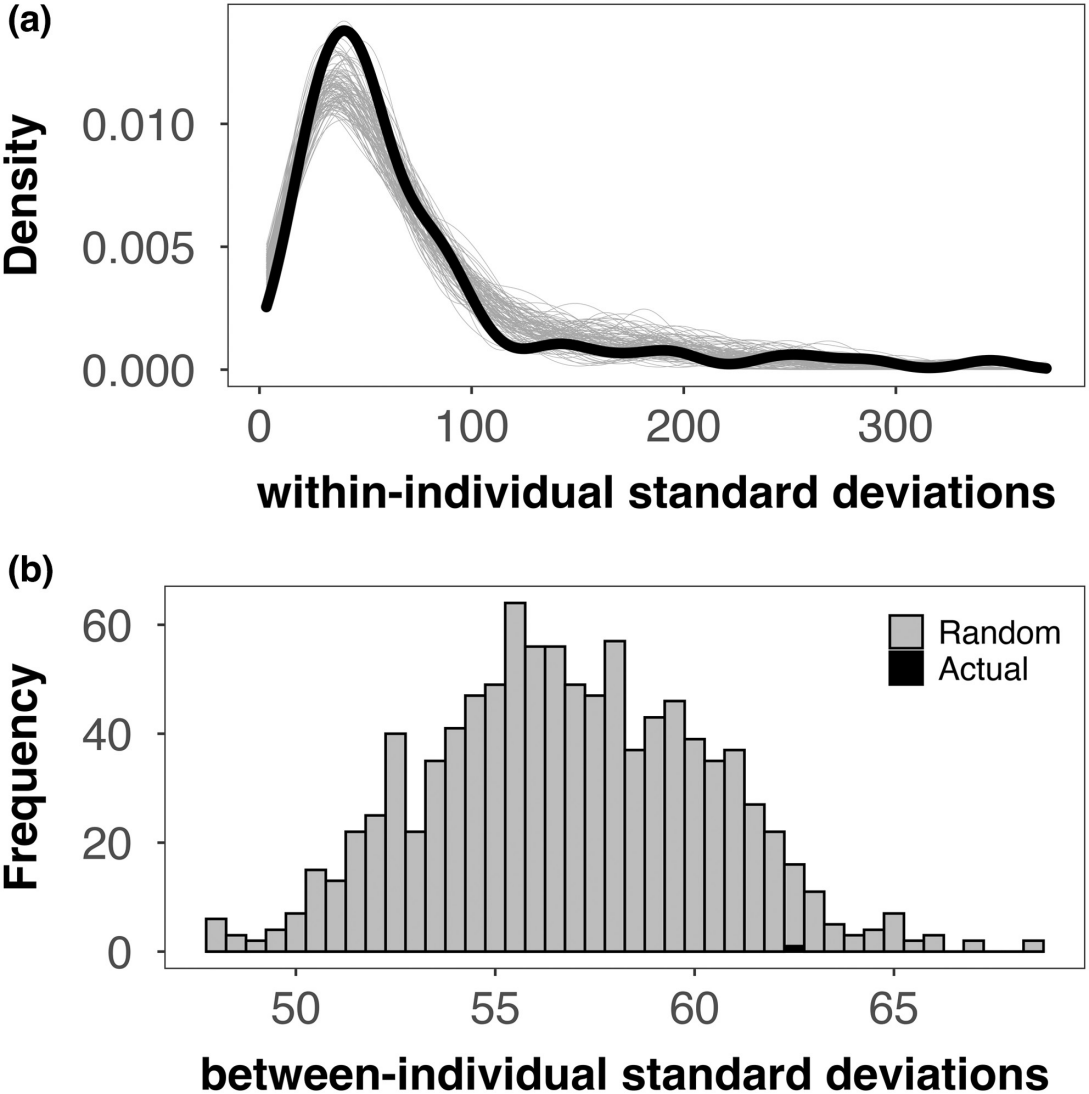
**(**a) Distribution of within‐individual standard deviations for 24 h displacement distances (*N* = 81) for the actual distribution (black) and for 1000 random‐derived values (gray). (b) Distribution of between‐individual standard deviation of standard deviations for random‐derived values (gray, *n* = 1000) and the actual value (black, *n* = 1).

We obtained 620 24 h displacement distances to assess the presence of movement personalities among 81 individual toads, with between 5 and 24 distances per toad. The distribution of the within‐individual standard deviations was right‐skewed (*x̃* = 35.99 m, *x̄* = 63.33 m; Shapiro–Wilk test: *W* = 0.83, *p* < .001; Figure 2a) and the calculated between‐individual standard deviation of these values was 62.23 (Figure 2b). As the between‐individual standard deviations of standard deviations derived from 1000 randomizations of the distance data were normally distributed (Shapiro–Wilk test: *W* = 0.98, *p* = .12) around a mean of 56.83, the actual value we obtained was not significantly different from a random result (Fisher's exact test: *p*_two‐tailed_ = .108; Figure 2b). The values resulting from the two, sorted, non‐random datasets used as a proof‐of‐concept test were 10.38 and 14.37, respectively, both far outside the distribution of randomized results (*p*_two‐tailed_ < .001).

## 4 DISCUSSION

Our results demonstrate that *A. fowleri* movements cannot be convincingly explained by any of the intrinsic or extrinsic variables that could be considered drivers of these movements. All variables we considered are too weakly correlated with movement distances to be biologically meaningful drivers of movement as they account for effectively none of the variations in distances moved by these animals. Thus, the 24 h movement distances of these toads are demonstrably neither sex‐biased, age‐biased, size‐biased nor density‐dependent. Nor are they correlated with environmental variables such as air temperature, precipitation, or lake water level, or demonstrate any consistencies indicative of distinct movement personalities. These findings are not unique as Deguise and Richardson (2009) obtained similar results for daily movements of Western Toads, *Anaxyrus boreas*, but in a fragmented landscape. The fine temporal and spatial scales of our measured movement distances, as well as the large size of our dataset, leave little room for noise in our statistical analyses.

Just as Smith and Green (2006) found, our data provide no convincing evidence for sex bias in movement distances among adult *A. fowleri* toads. Like most anuran amphibians (Kupfer, 2007), adult female *A. fowleri* average larger than adult males, and both average larger than sexually immature juveniles, resulting in a statistically significant, though weak, correlation between body size (i.e., SVL) and sex. This can make it difficult to distinguish between sex‐biased and size‐biased patterns of dispersive movements if sufficient data are not available for analysis. However, we amassed enough data from similarly sized adult males and females to conclude that sex is likely not a significant driver of movement distances. Greenwood (1980) states that the direction of the sex bias in dispersive movements is a consequence of the type of mating system. Thus, female‐biased dispersal should arise when males are highly territorial, as is generally seen in birds (Baker, 1978), whereas male‐biased dispersal would be selected for when females invest heavily in offspring and only breed once a year, as generally seen in mammals (Lidicker, 1975). These statements have been supported by research on amphibians (e.g., Beshkov & Jameson, 1980; Pilliod et al., 2002; Turner, 1960; Weintraub, 1974), but are also often refuted, with reports of either a lack of sex‐bias (e.g., Berven & Grudzien, 1990) or an unexpected direction of the bias in relation to the mating system (e.g., Lampert et al., 2003). In *A. fowleri*, males do not compete with females (Green, 1992) suggesting sexual selection is by female choice and dispersal should be male‐biased. Yet, we found no proof of male‐biased movement distances across spatial scales. Thus, there is not enough evidence that the mating system is sufficient to explain variation in movement distances, particularly in amphibians (Helfer et al., 2012). Instead, Trochet et al. (2016) report that sexual asymmetry in morphology and parental care seems to be the main driver of sex‐biased dispersal across species, opposing Greenwood's (1980) expectations.

Although the juvenile stage is often suggested to constitute the dispersive stage in many animals (Baker, 1978), particularly amphibians (Breden, 1987; Dole, 1971; Kupfer & Kneitz, 2000), we find no evidence for juvenile‐biased dispersal in *A. fowleri*. In amphibians, juvenile dispersal can be classified as natal dispersal, the movement of individuals from their birth site to their potential breeding site (Pittman et al., 2014). As most amphibian species display high post‐metamorphic mortality rates (Rothermel & Semlitsch, 2002; Semlitsch, 1981), juveniles, in theory, must disperse more or further than adults to acquire resources not already seized by adults (Smith & Green, 2006). However, the mortality rate in *A. fowleri* is high at all life stages and even varies from year to year due to high environmental variation (Middleton & Green, 2015). In addition, dispersal, survival, and recapture rates are often confounded, leading to biased estimates of dispersal rates and distances (Cayuela et al., 2020). Thus, the perception of juveniles as the dispersive stage is not necessarily linked to survival at this stage, but may be an artifact of the larger abundance of juveniles present in the population (Smith & Green, 2006).

The relationship between density and dispersal may be complex and possibly non‐linear—density‐dependence could only be apparent above a density threshold (Baines et al., 2014) or could switch from a negative to a positive correlation past a certain point (Fattebert et al., 2015; Kim et al., 2009). Testing for a density threshold has not yet been documented in amphibians, but the abundance of *A. fowleri* at Long Point is known to vary considerably from year to year (Greenberg & Green, 2013). As our population was not at a high enough density to designate a threshold, it was appropriate to use nearest‐neighbor distance, a measure typically used to assess nest dispersion (Clark & Evans, 1954), as a proxy for conspecific density. Nearest‐neighbor distance was significantly yet weakly correlated with all 24 h distances moved by *A. fowleri* and was no longer significant in the subsets for small and large spatial scales. Yet, whereas density has often been suggested to drive dispersal (Baguette et al., 2011; Clobert et al., 2004; Ronce, 2007), individual movements in relation to conspecifics within a population, might, conversely, drive density instead. If individual dispersive movements decrease the relative density, and aggregative movements do the opposite, then relative density is an outcome of movement rather than a driver. As the relationship between density and movement is two‐way, using nearest‐neighbor distance allows for a broader assessment of density, whereby density is best assessed based on the individual locations of the animals relative to one another rather than the unit area.

Unpredictable environmental variation can promote indeterministic movements in a manner similar to how seasonal, predictable variation in resource availability promotes migration (Jonzén et al., 2011). The positive correlation we observe between landscape variability and 24 h movement distances, although weak, is consistent with a hypothesis that environmental unpredictability resulting from stochastic landscape changes will influence animals' movement distances. Over the past two decades, Lake Erie water levels have been highly variable and have lately been at historic highs, in line with trends recorded in the other Great Lakes (Gronewold & Rood, 2019). A higher water level translates to a narrower beach and a disrupted dune structure in the Long Point landscape. But *A. fowleri* can locate previous refuge sites despite landscape dynamicity, as they can be home from various distances away from their starting point following artificial displacement (Jreidini & Green, 2022). Thus, unpredictable landscape change impacts *A. fowleri* stochastic movement distances, to some extent, but not by disrupting their homing capacities.

Individuals vary in behavioral expressions or syndromes, such as risk‐taking, boldness, activity, and exploration, where this variation is not necessarily attributable to sex, size, age, and state (Sih et al., 2004). A behavioral inconsistency between individuals, or “personalities”, has been reported in several studies on animal activity (Bell et al., 2009), but we find no evidence for consistent movement personalities in *A. fowleri*. To our knowledge, this is the first test of movement personalities in a terrestrial amphibian. With the surge in research investigating individual behavioral differences (Kelleher et al., 2018), particularly behaviors that have important implications for animal reintroductions and other conservation initiatives (Merrick & Koprowski, 2017), it becomes increasingly important to consider individuality in models of animal movement and dispersal (Fraser et al., 2001; Taylor & Cooke, 2014).

There exists a conceptual gap between the fields of animal movement ecology, as formulated by Nathan et al. (2008), and animal dispersal ecology, as conceived of by Clobert et al. (2004) and Ronce (2007), concerning dispersive movements. In the movement ecology literature, individual movements are generally proposed to be governed by random effects (Antman et al., 2001; Hanski, 1999; Tilman & Kareiva, 2018) whereas, in the animal dispersal literature, dispersive movements are commonly considered to be determined by drivers (Clobert et al., 2004; Denomme‐Brown et al., 2020; Matthysen, 2012). The *Anaxyrus fowleri* population at Long Point, Ontario, is not demonstrably driven to disperse (Marchand et al., 2017; Smith & Green, 2006). And yet they move. Our results from studying a very simple system of small terrestrial amphibians traveling at will to and fro along a lakeshore do not exclude the possibility that dispersal in other organisms in other environments may be significantly driven by any combination of internal and/or external variables. We do show, however, that this need not necessarily always be true.

## AUTHOR CONTRIBUTIONS

**Nathalie Jreidini:** Conceptualization (equal); data curation (equal); formal analysis (lead); methodology (lead); visualization (lead); writing – original draft (lead); writing – review and editing (equal). **David M. Green:** Conceptualization (equal); data curation (equal); formal analysis (supporting); methodology (supporting); supervision (lead); visualization (supporting); writing – review and editing (equal).

## FUNDING INFORMATION

This research was funded by grants from the Natural Sciences and Engineering Research Council (NSERC) of Canada, the Ontario Ministry of Natural Resources and Forestry, and the Ontario Ministry of Environment, Conservation and Parks to DMG.

## CONFLICT OF INTEREST

The authors declare no conflicts of interest.

## Data Availability

Data used in this study are available on the Dryad Digital Repository: https://doi.org/10.5061/dryad.0zpc86702.
